# Effect of an Electronic Health Record “Nudge” on Opioid Prescribing and Electronic Health Record Keystrokes in Ambulatory Care

**DOI:** 10.1007/s11606-020-06276-1

**Published:** 2020-10-26

**Authors:** Jessica S. Ancker, J. Travis Gossey, Sarah Nosal, Chenghuiyun Xu, Samprit Banerjee, Yuming Wang, Yulia Veras, Hannah Mitchell, Yuhua Bao

**Affiliations:** 1 Department of Population Health Sciences, Weill Cornell Medicine, New York, NY USA; 2 Physician Organization Information Services, Weill Cornell Medicine, New York, NY USA; 3 Department of Medicine, Weill Cornell Medicine, New York, NY USA; 4 Institute for Family Health, New York, NY USA

**Keywords:** opioid prescribing, electronic health records, electronic prescribing, EHR workload, clinical decision support, behavioral nudge

## Abstract

**Background:**

Multiple policy initiatives encourage more cautious prescribing of opioids in light of their risks. Electronic health record (EHR) redesign can influence prescriber choices, but some redesigns add to workload.

**Objective:**

To estimate the effect of an EHR prescribing redesign on both opioid prescribing choices and keystrokes.

**Design:**

Quality improvement quasi-experiment, analyzed as interrupted time series.

**Participants:**

Adult patients of an academic multispecialty practice and a federally qualified health center (FQHC) who received new prescriptions for short-acting opioids, and their providers.

**Intervention:**

In the redesign, new prescriptions of short-acting opioids defaulted to the CDC-recommended minimum for opioid-naïve patients, with no alerts or hard stops, such that 9 keystrokes were required for a guideline-concordant prescription and 24 for a non-concordant prescription.

**Main Measures:**

Proportion of guideline-concordant prescriptions, defined as new prescriptions with a 3-day supply or less, calculated per 2-week period. Number of mouse clicks and keystrokes needed to place prescriptions.

**Key Results:**

Across the 2 sites, 22,113 patients received a new short-acting opioid prescription from 821 providers. Before the intervention, both settings showed secular trends toward smaller-quantity prescriptions. At the academic practice, the intervention was associated with an immediate increase in guideline-concordant prescriptions from an average of 12% to 31% of all prescriptions. At the FQHC, about 44% of prescriptions were concordant at the time of the intervention, which was not associated with an additional significant increase. However, total keystrokes needed to place the concordant prescriptions decreased 62.7% from 3552 in the 6 months before the intervention to 1323 in the 6 months afterwards.

**Conclusions:**

Autocompleting prescription forms with guideline-recommended values was associated with a large increase in guideline concordance in an organization where baseline concordance was low, but not in an organization where it was already high. The redesign markedly reduced the number of keystrokes needed to place orders, with important implications for EHR-related stress.

**Trial Registration:**

www.ClinicalTrials.gov protocol 1710018646

**Electronic supplementary material:**

The online version of this article (10.1007/s11606-020-06276-1) contains supplementary material, which is available to authorized users.

## INTRODUCTION

One of the many contributing factors to the nationwide epidemic of opioid use and misuse^1^ has been high rates of prescribing of opioids at the population level.^2^ Almost all opioids involved in misuse or overdose originate from prescriptions,^3^ and prescription opioid misuse may have contributed to the rapid increase in heroin use^4^ and heroin and fentanyl fatalities.^5^ Although opioid therapy was once considered appropriate for chronic pain, newer evidence suggests long-term opioids have limited benefits^6–8^ and carry serious risks of misuse or opioid use disorder.^9^ Higher quantities and doses are associated with increased risk of overdose^6,7^ and, among opioid-naive patients, with higher risk of progression to long-term use.^10–12^

In 2016, the Centers for Disease Control and Prevention (CDC) published an influential Guideline for Prescribing Opioids for Chronic Pain^6,7^ that recommended (among other measures) starting opioid-naïve patients with acute pain on the lowest effective dose, generally 3–7 days’ supply. Multiple policy initiatives have also promoted lower-risk prescribing, ranging from prescription drug monitoring programs (PDMPs)^13^ to insurance coverage limitations.^14^

However, many of these initiatives complicate healthcare provider work by requiring them to log in to an external system such as a PDMP, to submit requests for pre-authorization, or to revise prescriptions after they have been rejected by an insurer or pharmacy. Electronic clinical decision support (CDS) systems that provide alerts or reminders about best practices can also complicate clinical work by interrupting work and requiring additional keystrokes or justifications for override decisions.^15^ Given that poor EHR usability is already recognized as a cause of physician stress and possible source of errors,^16,17^ initiatives that create more work in the EHR should be avoided.

Making it simpler to choose the recommended option can influence choices without requiring additional work. For example, organ donation rates are highest in countries where all individuals automatically have donor status but may opt out,^18^ and participation in 401(k) savings plans is highest when new employees are defaulted to participate.^19^ Electronic prescribing systems can exploit this “default effect” by automatically populating order forms with a recommended option, which has been demonstrated to change ordering choices in healthcare.^20–24^ Auto-completing forms also markedly reduces the number of keystrokes needed to perform recommended actions, which could reduce the keystroke burden in contemporary EHRs.^25^

The objective of the current project was to test the effect of a default prescription order intervention on opioid prescribing choices, with a secondary outcome of number of keystrokes needed to write the order. Previous studies of similar interventions^20–24^ have used pre-post designs, but given the known nationwide trends toward reduced opioid prescribing,^26^ it seems likely that a pre-post design would overstate the size of the effect by conflating the secular trend with the intervention effect. We therefore used an interrupted time series analysis to account for concurrent secular changes. We assessed the intervention in two ambulatory settings, an academic multispecialty practice and a federally qualified health center (FQHC) providing safety net care.

## METHODS

### Overview

A quality improvement study was conducted using quasi-experimental methods. The study was approved by the institutional review boards of Weill Cornell and the Institute for Family Health and registered at ClinicalTrials.gov as protocol 1710018646.

### Settings

Weill Cornell Medicine is the faculty practice of Weill Cornell Medical College in New York City. The organization includes a variety of specialty practices and hospital-based clinics that include residents. The Institute for Family Health (IFH) is a federally qualified health center providing primary care to a safety net population at 31 practice sites in and around New York City. Most IFH providers are family practice physicians and advanced practice nurses. IFH also trains family practice residents as part of Mount Sinai’s Icahn School of Medicine. Both Weill Cornell and IFH use the Epic electronic health record.

### Intervention

The intervention was applied to new prescription orders for short-acting opioids in pill or tablet form for adult patients. When a new prescription order was opened and the prescriber typed in the drug, the order auto-completed to a quantity of 12 pills or tablets and a frequency of 4 times per day (3-day supply). The prescriber could type over the default without providing any justification. Before the intervention, prescription order form fields for opioids were blank (at the FQHC) or defaulted to 7 days’ or 30 days’ supply or blank fields (at the academic medical center). The redesign standardized all short-acting opioids so that 9 keystrokes were needed to write a guideline-concordant prescription, compared to 24 for a non-concordant prescription.

No similar intervention was put into place for long-acting opioids, as they are typically used for different indications such as cancer, in which a 3-day supply might not be optimal. Prescriptions placed using the prescription renewal option within the electronic prescribing system were also excluded, because the CDC Guideline recommendation of interest was the one for opioid-naïve patients.

The intervention was implemented in March 2018 at the academic medical center, and it was replicated at the FQHC in July 2018. At both sites, the change was preceded by a system-wide email announcement that explained its grounding in the CDC Guideline.

### Participants

Participants include adult patients who received a new prescription for a short-acting opioid between January 1, 2015 and December 31, 2018, together with the providers who prescribed them. Patients who received care from cancer specialties or chronic pain clinics were excluded from the analysis.

### Measures

The primary outcome was proportion of short-acting opioid prescription orders placed as a new order that contained 12 pills or fewer, i.e., a 3-day supply (“guideline-concordant prescriptions”). To construct the outcome measure, we identified all new opioid prescription orders, and additionally confirmed that the patient had no opioid prescription orders in the EHR in the previous 12 months.

Prescribing data were retrieved from both sites and proportions of guideline-concordant prescriptions were calculated at 2-week intervals. Because the primary analysis was at the prescription level, a patient could appear in the data set more than once.

For the keystroke estimates, two informatics leads (YV, SN) walked through the e-prescribing workflow to count the number of clicks and typed letters or words needed per prescription with and without the default intervention. Before the intervention, all IFH opioid prescriptions defaulted to blank fields and required 24 keystrokes, and after the intervention, all concordant prescriptions required 9 keystrokes. Estimates were calculated by multiplying numbers of concordant and non-concordant prescriptions by the appropriate number of keystrokes. However, before the intervention at the academic medical center, default quantities varied, and no reliable estimate of the pre-intervention keystrokes could be calculated.

Additional secondary outcomes were proportions of new prescription orders containing (a) 3 to 7 days’ supply and (b) more than 7 days’ supply.

### Statistical Analysis

For the primary analysis, we applied segmented regression to conduct interrupted time series analysis (ITS), which is appropriate for quasi-experimental designs because it allows estimation of the pre-intervention trend (slope of the regression line), the immediate effect associated with the intervention (change in regression line intercept), and any change in trend after the intervention (change in slope). ITS thus accounts for secular trends that could confound simple pre-post designs.^27–29^ We considered this important for the current study because of the known concurrent secular trends in opioid prescribing and, in particular, the initiation of multiple local, state, and national policies and initiatives influencing opioid prescribing.

The ITS model estimated changes in the intercept and slope of the data series at two interruptions established a priori: release of the CDC Guideline in March 2016 and the default intervention (March 2018 for the academic medical center, July 2018 for the FQHC). After running a Durbin-Watson test to confirm that there was no significant autocorrelation, estimates of intercepts and slopes of the primary outcome were obtained using linear regression. The models estimated changes in intercept and slope of the primary outcome associated with publication of the CDC Guideline in (first interruption) and with the electronic prescribing intervention (second interruption). A seasonality term was found to be non-significant and was dropped from the final models. Calculating the proportion of concordant prescriptions every 2 weeks resulted in a minimum of 12 data points before and between the two events of interest: this sample size is recommended as minimum for ITS.^29^ Analyses were stratified by site. As a post hoc analysis, we also conducted a stratified analysis at the academic medical center by provider type (medical versus surgical).

## RESULTS

The academic medical center had 18,218 patients meeting inclusion criteria, versus 3895 at the FQHC (Table 1). The large majority (77%) of WCM patients were commercially insured, whereas the most prevalent insurance type at the FQHC was Medicaid, covering 44% of included patients. There were very few uninsured patients in the samples because prescription drug assistance programs for uninsured patients do not typically cover controlled substances.

**Table 1:** **Characteristics of Patients Receiving Short-Acting Opioid Prescriptions and of Their Providers at the Two Study Sites, January 1, 2015–December 31, 2018**

	*N* (%)^1^	
	Weill Cornell Medicine	Institute for Family Health
Total patients	18,218 (100.0)	3895 (100.0)
Number female	9139 (49.4)	2705 (68.6)
Number white	3562 (19.2)^4^	1639 (41.6)
Number commercially insured^2^	7638 (77.2)	2524 (25.6)
Number Medicaid	149 (1.5)	4261 (44.2)
Number Medicare	2106 (21.3)	2866 (29.0)
Number uninsured	0 (0.0)	110 (1.0)
Total prescribers	585 (100.0)	236 (100.0)
Attending physician (MD or DO)	417 (71.3)	192 (81.4)
Resident or fellow	13 (2.2)	45 (19.1)
Advanced practice nurse (FNP/ANP) or PA	103 (17.6)	39 (16.53)
Prescriber specialty		
Internal medicine^3^	72 (12.31)	4 (1.7)
Family practice	0 (0)	162 (68.6)
Other medical	242 (41.38)	70 (26.9)
Surgical	271 (46.32)	0 (0)
Total new short-acting opioid prescriptions	18,518 (100.0)	3943 (100.0)
Internal medicine	1666 (9.0)	160 (4.1)
Family practice	0 (0)	3413 (86.5)
Other medical	6170 (32.3)	370 (9.4)
Surgical	10,682 (57.7)	0 (0)

^1^Counts of patients and providers reflect unique patients and providers even though each patient and provider could appear in the data set more than once

^2^For simplicity, the distribution of insurance types reflects the last covered visit in 2018

^3^At the academic medical center, “internal medicine” includes primary care as well as internal medicine subspecialties such as cardiology and endocrinology. “Other medical” includes emergency medicine, rehabilitation medicine and physical therapy, psychiatry, dialysis, and (non-interventional) radiology

^4^At the academic medical center, 59.9% of patients (11,091) had unknown race

At the academic medical center, medical specialties accounted for 53% of opioid prescribers and 41% of prescriptions (Table 1). Surgical specialties accounted for 46% of all opioid prescribers and 58% of all opioid prescriptions. By contrast, almost all opioid prescribers at the FQHC were in family practice (87%) and internal medicine (4%), and these primary care specialties accounted for more than 90% of opioid prescriptions.

At both sites, proportion of guideline-concordant prescriptions rose consistently from early 2016 until the intervention in 2018 (. 1). At the time of the intervention (vertical red line), the academic medical center’s rate of concordant prescribing averaged approximately 12%, and the FQHC’s averaged approximately 44%. The intervention was associated with an immediate increase in guideline-concordant prescribing at the academic medical center, more than doubling the concordance rate. By contrast, at the FQHC, the scatterplot does not show discernible immediate change.

**Figure 1:**
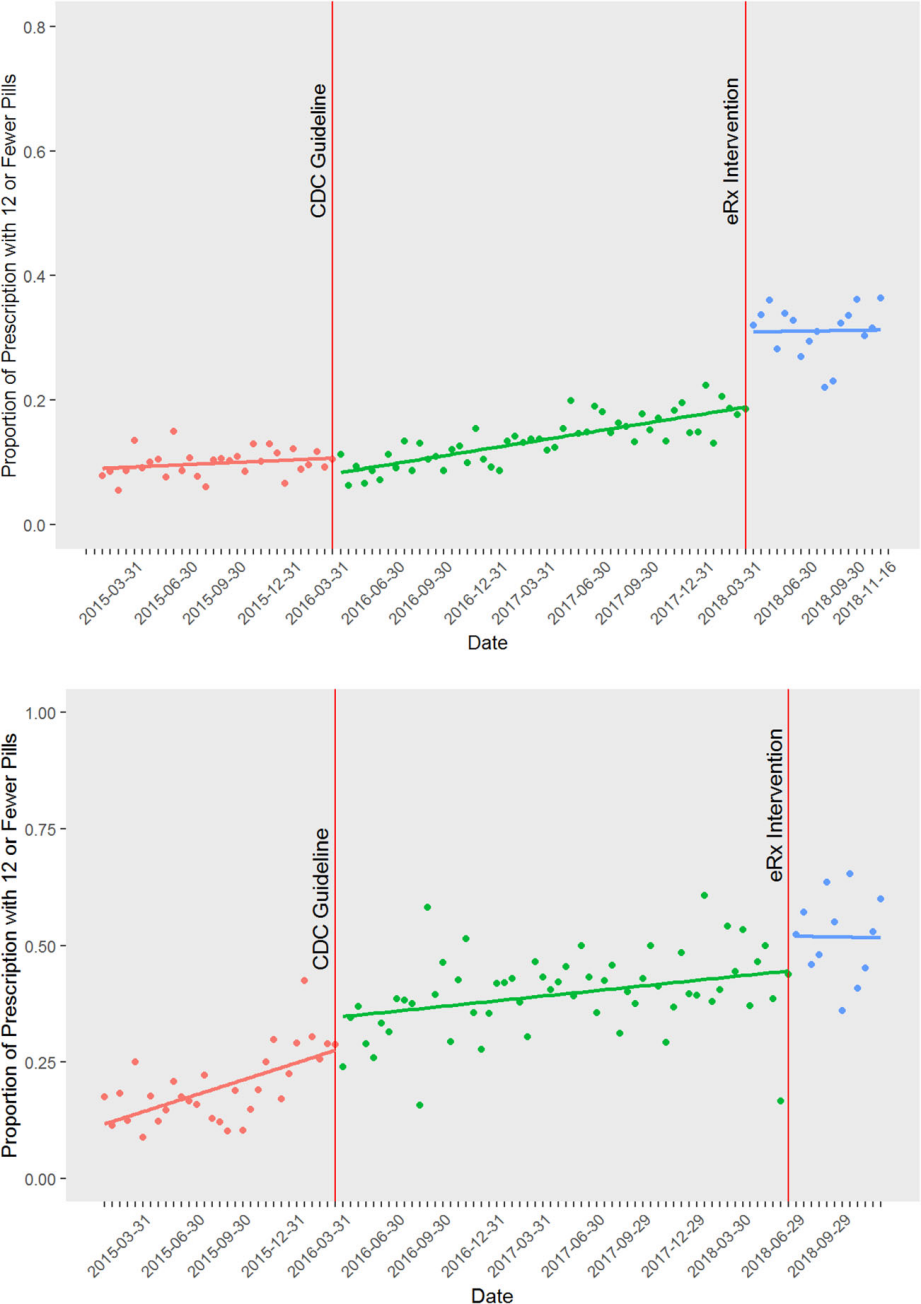
**Rates of guideline-concordant prescribing per 2-week interval at the academic medical center (top) and the FQHC (bottom). The vertical red line in March 2016 indicates the publication of the CDC Guideline, treated as interruption 1 in the interrupted time series models. The vertical red line in March 2018 (the academic medical center) and June 2018 (the FQHC) indicates the default intervention, treated as interruption 2 in the models. Superimposed trend lines were constructed using segmented regression. Note that for the FQHC, the small increase at the time of the eRx intervention was found to be non-significant.**

**Figure 2:**
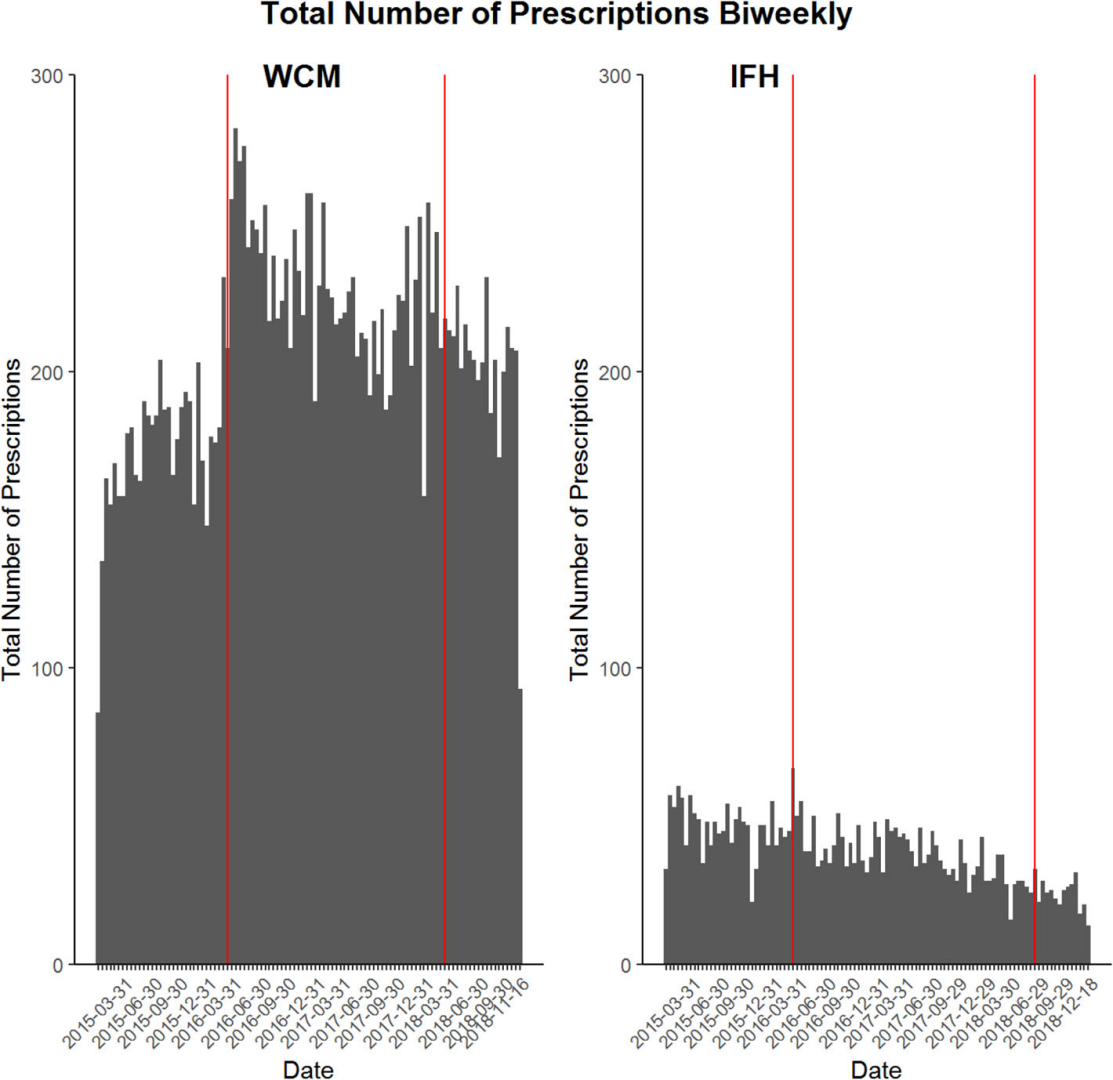
**Total numbers of opioid prescriptions per 2-week interval at the academic medical center (left) and the FQHC (right). As in Figure** **1****, the vertical red line in March 2016 indicates the publication of the CDC Guideline, and the line in March 2018 (the academic medical center) and June 2018 (the FQHC) indicates the default intervention.**

The intervention was not associated with any change in total volume of opioid prescriptions, which had been decreasing steadily since early 2016 at both sites (Fig. 2).

**Figure 3:**
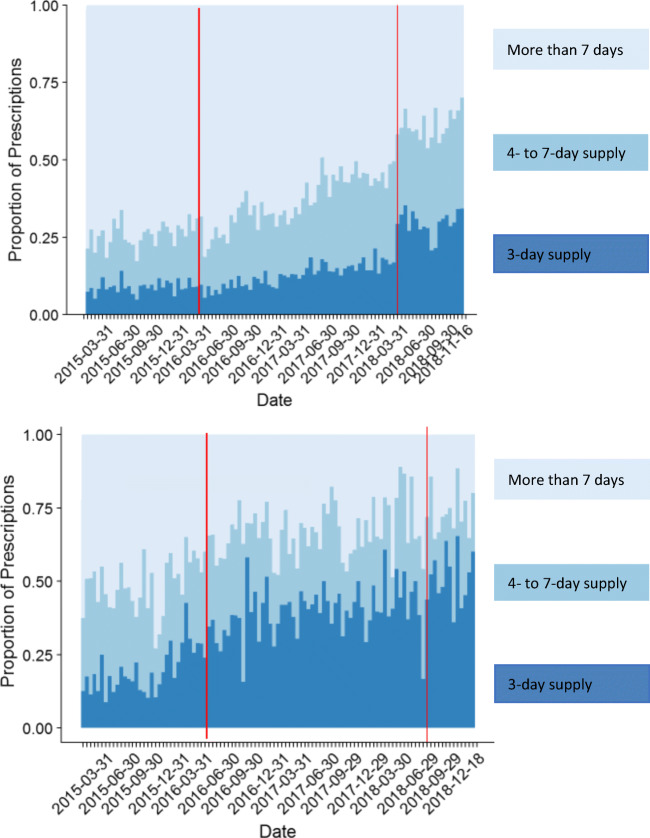
**Distribution of quantities of short-acting opioid prescriptions at the academic medical center (top) and the FQHC (bottom). As in Figure** **1****, the vertical red line in March 2016 indicates the publication of the CDC Guideline, and the line in March 2018 (the academic medical center) and June 2018 (IFH) indicates the default intervention.**

At both sites (Fig. 3), the proportion of prescriptions with more than a 7-day supply (more than 28 pills) also decreased at each site over time. At the academic medical center, the proportion of these longer prescriptions fell abruptly at the intervention.

The interrupted time series estimates (Table 2) show that the publication of the CDC Guideline was associated with prescribing changes at both sites, but the default intervention was associated with significant changes only at the academic medical center.

**Table 2:** **Interrupted Time Series Estimates of Effects of CDC Guideline Publication and e-Prescribing Intervention**

Site	Term	Estimate	Confidence interval	*p*
Academic medical center	(Intercept)	0.089	0.068, 0.109	< 0.001
	Time (biweek)	0.0006	− 0.0005, 0.002	0.28
	CDC (interruption 1)	− 0.025	− 0.049, − 0.0004	0.049
	Change in slope after CDC	0.0015	0.0003, 0.0027	0.01
	eRx (interruption 2)	0.112	0.085, 0.139	< 0.001
	Change in slope after eRx	− 0.001	− 0.004, 0.002	0.435
Federally qualified health center	(Intercept)	0.107	0.048, 0.166	< 0.001
	Time (biweek)	0.005	0.002, 0.008	0.001
	CDC Guideline (interruption 1)	0.067	− 0.003, 0.136	0.06
	Change in slope after CDC	− 0.004	− 0.007, 0.0003	0.006
	eRx intervention (interruption 2)	0.073	− 0.02, 0.166	0.13
	Change in slope after eRx	− 0.002	− 0.015, 0.011	0.76

Specifically, at the academic medical center, the proportion of guideline-concordant prescriptions remained flat over time before the publication of the Guideline (i.e., the term for “biweekly period” was non-significant). The Guideline was associated with an immediate 2 percentage point decrease in concordant prescribing (*p* = 0.049), followed by a significant increase in slope reflecting a novel upward trend of 0.15% per biweekly period, i.e., 3.6% per year (*p* = 0.01). The e-prescribing intervention was associated with an additional immediate 11 percentage point increase in concordant prescribing (*p* < .001), with no significant change in slope, showing that the upward trend was not altered at this time. Stratified analyses show very similar effects among surgical and medical specialties. That is, the e-prescribing intervention was associated with an 11.5 percentage point increase in guideline-congruent prescribing among medical providers and an 11.7 percentage point increase among surgical providers (both *p* < 0.001; Appendix 1 provides the scatterplots and models).

At the FQHC, proportion of guideline-concordant prescriptions was rising before the CDC Guideline at a rate of 0.5% per biweekly period (or 12% per year). The Guideline was associated with a small increase in concordant prescribing that was not statistically significant and with a very small but significant decrease in slope, such that the overall slope remained positive but there was a small deceleration at that time. At the FQHC, the e-prescribing intervention was associated with a 7 percentage point increase in concordant prescribing, but this was not statistically significant. There was also no significant change in slope, meaning that there was no immediate increase and that the previous upward trend in concordant prescribing remained unchanged.

At the FQHC, over the 6 months before the intervention, prescribers wrote 148 guideline-concordant prescriptions using a total of 3552 keystrokes, and over the same time period after the intervention wrote almost the same number (147 concordant prescriptions). However, the total number of keystrokes required for these dropped to 1323, a 62.7% decrease.

At the academic medical center, prescribers wrote 455 guideline-concordant prescriptions over the 6 months before the e-prescribing intervention, but the different default quantities meant total number of keystrokes could not be estimated. In the subsequent 6 months, the number of guideline-concordant prescriptions rose 63% to 741, and at 9 keystrokes each, keystrokes totaled 6669.

## DISCUSSION

An unobtrusive “nudge” involving changing the default option on an electronic prescribing interface was associated with an immediate increase in prescribing concordant with national recommendations, but only in a setting where rate of guideline concordance was low at baseline. It had a smaller and non-significant effect in a setting where guideline-concordant prescribing was already prevalent, suggesting a ceiling effect. The effect associated with the intervention remained evident even after controlling for marked secular trends toward reduced opioid prescribing. The effect was similar among medical prescribers and surgical ones. The intervention did not add extra work for prescribers. Instead, it markedly reduced the number of keystrokes needed for concordant prescriptions. As a result, the total number of keystrokes expended for prescribing decreased, even at the site where there was no change in proportion of guideline-concordant prescriptions.

Reducing keystrokes has important implications for EHR-related burden.^30^ Electronic health records are recognized as a source of stress for healthcare providers, who blame factors including heavy data entry requirements, inability to navigate nimbly, and discomfort from typing and posture.^16,25,31^ Electronic clinical decision support systems (CDSS), which provide alerts and reminders to promote recommended practices, may contribute to EHR-related stress and dissatisfaction by requiring additional work, more keystrokes, and workflow interruptions.^15^ CDSS alerts are also becoming less effective because the large majority are currently overridden,^32–34^ and their high volume and poor specificity contribute to alert fatigue.^32,35^ The approach studied in this paper, by contrast, simplifies EHR work by reducing the number of keystrokes needed for recommended prescribing. As a result, the easier choice coincides with the recommended one. The 63% reduction in keystrokes we observed is nearly as large as the change associated with implementing predefined order sets in a recent study.^25^

Evidence from other sources suggests that the default is not only easier to choose but is also perceived to be endorsed by the architects of the social or technical system.^36^ As a result, the default effect influences decision-makers in domains as different as personal savings, postmortem organ donation, and end-of-life care preferences.^18,37^ In healthcare, pre-post studies have found that changing the electronic ordering default is associated with increased proportions of generics prescribed^20,21^ and reduced opioid prescribing in the emergency department.^24^

Implementing the same intervention in two very different ambulatory settings revealed differences in the effect on prescribing decisions. The default effect was substantial among medical providers in the academic medical center, where the rate of guideline concordance was low at baseline. The low concordance rate could have been the result of a diverse patient population treated by a multispecialty practice. The intervention was still associated with a large increase in concordant prescribing, presumably due to patients for whom the CDC Guideline–recommended quantities appeared appropriate.

By contrast, the intervention had no detectable effect at the FQHC, where clinicians providing primary care were already following CDC Guideline recommendations at a high rate. Figure 1 and the ITS analysis suggest that concordant prescribing at the FQHC was rising consistently both before and after the default intervention. An analysis that did not control for the secular trend toward improved guideline concordance might have mistakenly concluded that the intervention had an effect here as well. (Nevertheless, the intervention substantially reduced keystroke burden, even without an effect on prescribing choices.)

The study is limited by lack of a concurrent control group. However, the abrupt secular change noted at the academic medical center did not coincide with any known local or national policy initiatives, nor with any concurrent event evident in the FQHC data. Furthermore, the ITS analysis allowed us to estimate concurrent secular trends. The study is also limited by lack of data on clinical appropriateness of duration or dosage of opioid prescriptions, pain-related outcomes, satisfaction, or other patient outcomes. We identified prescriptions as new if they were placed using the new order functionality and limited the analysis to patients with no history of opioids at their care site in the previous 12 months. However, we had access only to data from each site’s EHR, not to PDMP or payer data that would include prescriptions from other locations, meaning the samples might have included some patients who were not opioid-naïve. If some of the new medication orders were placed for patients already receiving opioids (for example, if they were being switched from one medication to another), a 3-day prescription might have been considered less clinically appropriate, which could explain some of the non-concordant prescriptions. For these reasons, the optimal proportion of 3-day prescriptions, especially among the academic medical center’s multispecialty patient population, is not known. Finally, the two sites were extremely different, with the academic medical center serving a primarily commercially insured population, and the FQHC serving a publicly insured one.

We conclude that an unobtrusive “nudge” involving changing the default option was associated with an increase in guideline concordance in a setting with low baseline concordance, but not in a different setting where concordance was already high. The default intervention also drastically reduced the number of keystrokes needed to prescribe medications, which could affect EHR-associated stress.

## Electronic Supplementary Material

ESM 1 (PDF 147 kb)
