# Supplementary figures and images for: Effect of an Electronic Health Record “Nudge” on Opioid Prescribing and Electronic Health Record Keystrokes in Ambulatory Care
Source: J Gen Intern Med. 2020 Oct 26;36(2):430–7. doi: 10.1007/s11606-020-06276-1 (PMC7878599; doi:10.1007/s11606-020-06276-1)

Figure 1 WCM - Surgical

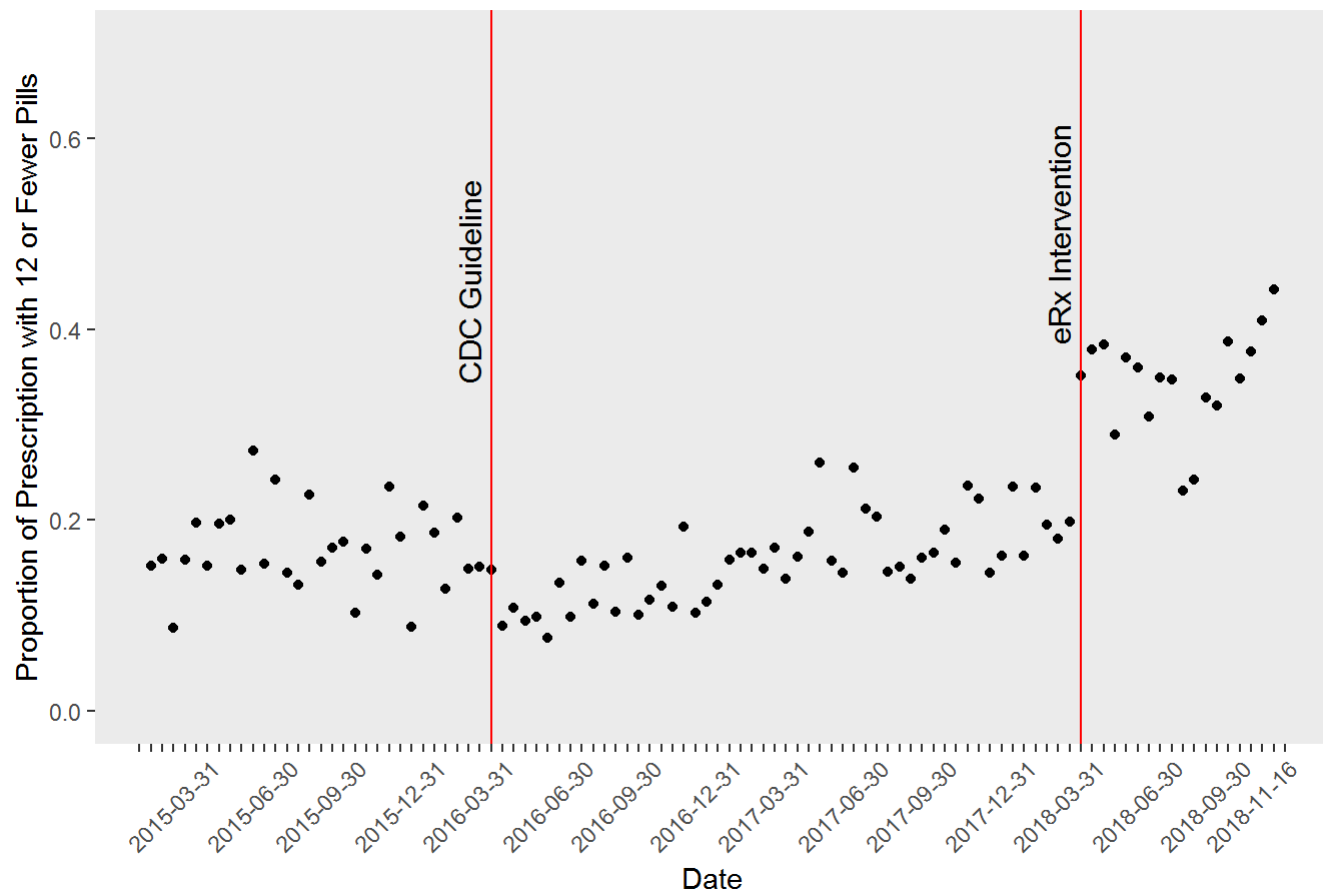

Figure 1 WCM - Medical

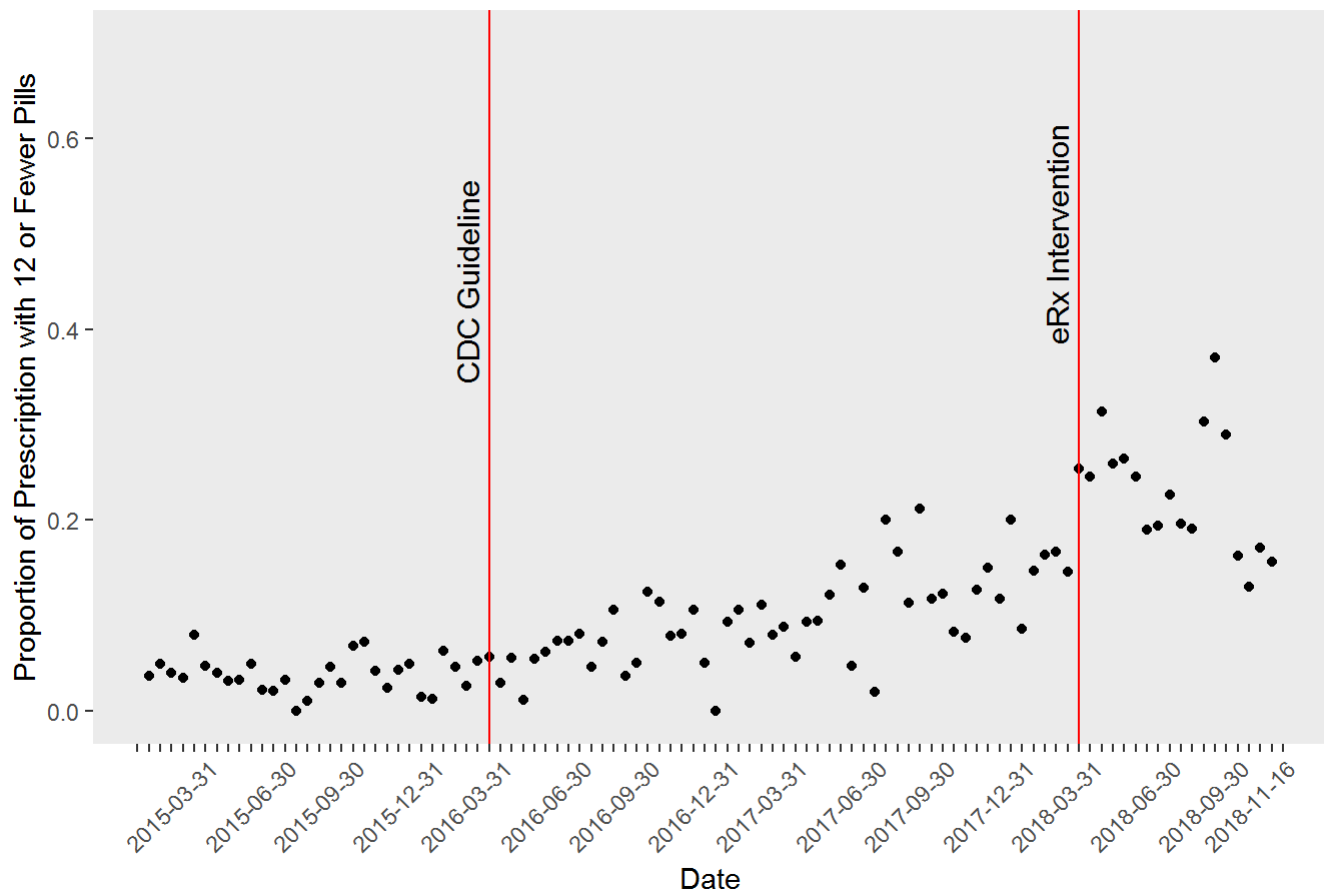

Supplement: Supplementary file 1 — (PDF 147 kb) [file 11606_2020_6276_MOESM1_ESM.pdf]
